# BSA-Seq and Transcriptomic Analysis Provide Candidate Genes Associated with Inflorescence Architecture and Kernel Orientation by Phytohormone Homeostasis in Maize

**DOI:** 10.3390/ijms241310728

**Published:** 2023-06-27

**Authors:** Yang Wang, Yang Li, Wenjie Zhang, Yue Yang, Yuting Ma, Xinyang Li, Dexuan Meng, Haishan Luo, Wei Xue, Xiangling Lv, Fenghai Li, Wanli Du, Xiaolin Geng

**Affiliations:** 1 Specialty Corn Institute, College of Agronomy, Shenyang Agricultural University, Shenyang 110866, Chinadxmeng@syau.edu.cn (D.M.);; 2 College of Agronomy and Biotechnology, China Agricultural University, Beijing 100193, China

**Keywords:** maize, inflorescence differentiation, reversed kernel, giant embryo, hormone homeostasis

## Abstract

The developmental plasticity of the maize inflorescence depends on meristems, which directly affect reproductive potential and yield. However, the molecular roles of upper floral meristem (UFM) and lower floral meristem (LFM) in inflorescence and kernel development have not been fully elucidated. In this study, we characterized the *reversed kernel1* (*rk1*) novel mutant, which contains kernels with giant embryos but shows normal vegetative growth like the wild type (WT). Total RNA was extracted from the inflorescence at three stages for transcriptomic analysis. A total of 250.16-Gb clean reads were generated, and 26,248 unigenes were assembled and annotated. Gene ontology analyses of differentially expressed genes (DEGs) detected in the sexual organ formation stage revealed that cell differentiation, organ development, phytohormonal responses and carbohydrate metabolism were enriched. The DEGs associated with the regulation of phytohormone levels and signaling were mainly expressed, including auxin (IAA), jasmonic acid (JA), gibberellins (GA), and abscisic acid (ABA). The transcriptome, hormone evaluation and immunohistochemistry observation revealed that phytohormone homeostasis were affected in *rk1*. BSA-Seq and transcriptomic analysis also provide candidate genes to regulate UFM and LFM development. These results provide novel insights for understanding the regulatory mechanism of UFM and LFM development in maize and other plants.

## 1. Introduction

The developmental activity of maize (*Zea mays* L.) inflorescence determines inflorescence architecture and thereby directly affects the reproductive potential and yield of this crop via a complex process involving multiple genes. Maize inflorescence development also has important effects on kernel formation [1]. Maize is monoecious, with two types of unisexual inflorescences located in different parts of the plant: the male tassel and the female ear [2]. During maize flower development, the inflorescence meristems (IMs) of the tassel and ear develop from shoot apical meristems (SAMs) and axillary meristems (AMs), respectively [3]. During tassel development, IMs initiate branch meristems (BMs). Subsequently, IMs and BMs successively initiate spikelet pair meristems (SPMs), which give rise to two spikelet meristems (SMs). The SMs gradually differentiate into the upper floral meristem (UFM) and lower floral meristem (LFM), which develop into the upper florets (UFs) and lower florets (LFs), respectively [4]. 

The development of the ear inflorescence is similar to that of the tassel inflorescence, except that no BMs are formed [5]. During floret development, the UFM develops slightly earlier than the LFM. Each FM forms floral organs: the inner and outer lemma, three stamen primordia, a pistil primordia, two lodicules, and a palea. In the tassel spikelet, the stamen primordia are eventually converted into anthers, while the pistils of the UF and LF successively abort after initiation [6]. In the ear spikelet, the pistil primordia of the UF initiate rapidly and develop, whereas the stamens of the UF gradually abort. Subsequently, each pistil of the UF produces an ovule with a silk, and the LF degenerates [6]. Thus, tassel florets develop into unisexual male florets, and the UFs of the ear develop into unisexual female florets upon maturation [3,6,7].

Maize inflorescence development is controlled by multiple genetic pathways and other factors. Many mutations can cause abnormal ear development, where extra florets develop and the orientation of the florets is altered [8]. During the development of the pistillate inflorescence, the SMs produce two florets. However, the *indeterminate spikelet1* (*ids1*) mutant produces extra florets and exhibits a reversed germ orientation with a disturbed rowing phenotype, indicating that *ids1* is critical for the regulation of SM development in maize [9,10]. Similarly, in *reversed germ orientation1* (*rgo1*) mutants, which were first described in 1980, the embryo faces the stalk end in many kernels of mutant ears. This reversal is caused by differences in the growth of the integuments and nucellus, which changes the direction of the ovule by 90° [11]. Kaplinsky and Freeling have shown that *rgo1* behaves as a single recessive mutant [10]. The visible phenotypes of *rgo1* include increased flower number in tassel and ear spikelets, irregular rows of kernels in the ear, and kernels with embryos facing both the tip and base of the ear. These phenotypes are due to the production of an extra floret by the SM [10]. The relationship between *ids1* and *rgo1* is non-allelic and non-complementing as the *rgo1 ids1* double mutant exhibits synergistic changes, with more severe phenotypes than either single mutant [8,10]. 

In summary, maize inflorescence development is shaped by the differentiation and formation of the IM. Thus, mutations affecting any stage of IM development may alter the establishment of the pistillate and staminate inflorescences. Although IM and SM mutants were shown to be affected in inflorescence and kernel development in maize, whether or how the order of UFM and LFM development might contribute to inflorescence and kernel development is largely unknown.

Flower differentiation is controlled by the sequential expression of specific genes. Phytohormones also have extremely important regulatory effects on inflorescence development. In particular, phytohormones such as auxin (e.g., indole-3-acetic acid [IAA]), gibberellin (GA), cytokinin (CTK), and abscisic acid (ABA) function together to regulate plant proliferation, development, and maturation [12]. IAA is an important plant hormone regulating many growth and developmental processes, including stem and root elongation and flower and fruit development [13]. Initiation of branch meristems with floral identity on the flanks of the inflorescence meristem is dependent on the auxin maximum at branch anlagen [14,15]. YUCCA proteins, which are involved in auxin biosynthesis, function in maize ear differentiation by regulating the expression of genes in the leaf and floral organ primordia in the spikelet [16]. 

GA is another plant hormone that affects sex determination in maize, where increased endogenous GA levels in tassels repress the formation of stamens and promote the development of pistils to ultimately result in the tassel-seed phenotype [17]. Floret differentiation is inhibited unless optimal GA levels are achieved. Once these levels are exceeded, reduced inflorescence fertility will appear [18]. The anthers and lower florets in pistillate inflorescences are not properly aborted in many mutants, such as the GA-related *anther ear1* (*an1*) and *dwarf plant8* (*d8*) mutants, leading to fewer long branches in the tassels, though the anthers and lower florets develop normally in the mutant ears [19,20]. 

In addition, CTKs, such as zeatin riboside, regulate sexual development in maize [21,22]. Young has studied the roles of the senescence-associated genes (*SAGs*) involved in CTK accumulation during maize floret development and determined that CTK inhibits the abortion of inferior floret pistils during ear differentiation, thereby affecting pistil development [22]. 

ABA plays important roles in the growth and development of various plant tissues and organs, such as the promotion of seed dormancy, inhibition of germination [23], stomatal closure [24], transmission of stress information [25], and fruit ripening and senescence [26]. Lejeune has suggested that ABA affects the fertility of florets during maize ear differentiation, thereby regulating the number of kernels in the ear [27]. In maize *ts1*, *ts2*, and *ts5* mutants, the pistils fail to abort in the tassel florets and lower florets of the ears, yielding feminized tassels and irregular kernel rows. This finding points to the involvement of the jasmonic acid (JA) biosynthesis and metabolism pathways, since *Ts1* encodes a lipoxygenase involved in JA biosynthesis, whereas *Ts2* and *Ts5* encode a short-chain alcohol dehydrogenase and CYP94B enzyme, respectively [28,29,30]. 

Endogenous phytohormones are normally in a dynamically balanced state in plants. Phytohormones promote, antagonize, or jointly affect the growth and development of plants. According to the hormone balance hypothesis, plant hormone levels fluctuate dynamically during inflorescence differentiation [31,32].

We previously identified a novel waxy corn line with reversed kernels and a giant embryo oriented toward the petiole. Large/macro-embryo traits have been reported in many crops, including maize [33], barley (*Hordeum vulgare*) [34], rice (*Oryza sativa*) [35], and scarlet runner bean (*Phaseolus coccineus*) [36], but the regulatory pathways and developmental mechanisms of reversed kernels and macro-embryos are largely unknown. 

In this study, we characterized the maize *reversed kernel1* mutant *rk1*, a novel mutant impaired in inflorescence development. We determined that the reversed kernel phenotype of *rk1* is due to the reversed order of UFM and LFM development. The mutant phenotype of *rk1* in the ear only occurs when *rk1* is transmitted through the female parent. *rk1* influences organ differentiation, phytohormone homeostasis, and nutrient contents. These findings increase our understanding of the molecular mechanisms that underlie inflorescence development in maize. A macro-embryo can store more nutrients than the wild type and improve seed vigor. Thus, *rk1* represents an important resource for studying inflorescence development, increasing the available germplasm resources, and facilitating the discovery and functional analysis of important genes in maize. 

## 2. Results

### 2.1. The Reversed Kernel1 Phenotype Affects Both Ear and Tassel Development

**Figure 1:**
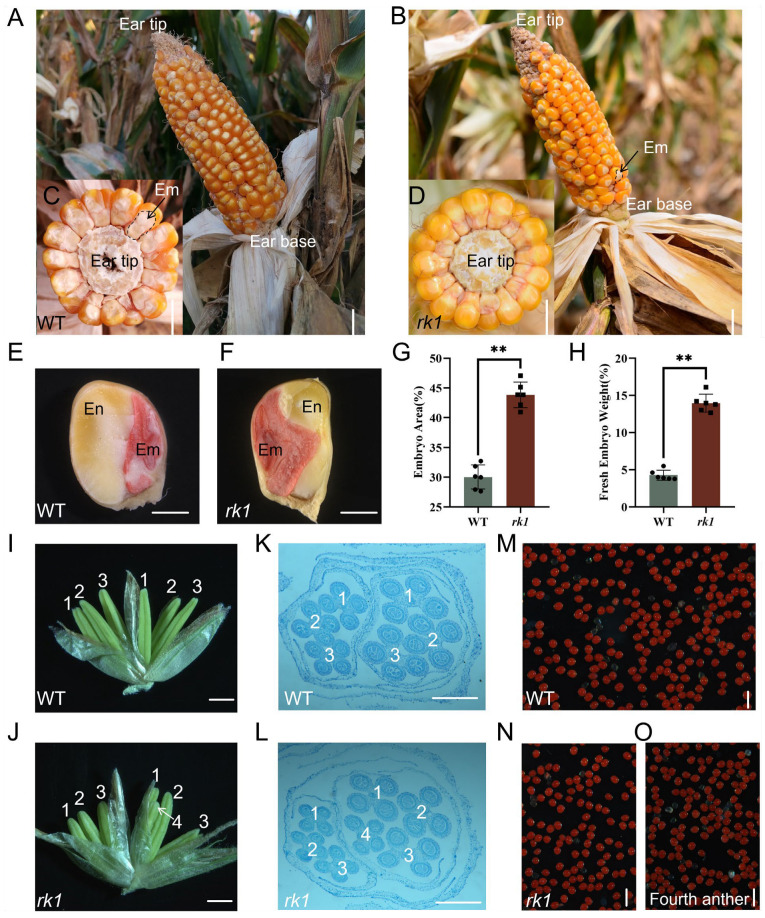
Morphological analysis of wild-type and *rk1* plants. (**A**) The grain embryo is oriented toward the tip of the ear in the wild type (WT). Scale bar = 1 cm. (**B**) The grain embryo is oriented toward the peduncle of the ear in *rk1.* Em, embryo. Scale bar = 1 cm. (**C**) WT embryos are clearly visible in an ear cross section. Em, embryo. Scale bar = 6 mm. (**D**) *rk1* embryos are not visible in an ear cross section. Scale bar = 6 mm. (**E**) Longitudinal sections of mature WT kernels. En, endosperm; Em, embryo. Scale bar = 2.5 mm. (**F**) Longitudinal sections of mature *rk1* kernels. En, endosperm; Em, embryo. Scale bar = 2.5 mm (**G**) Area of the embryo in WT and *rk1* kernels. Values are means ± SD (*n* = 6; ** *p* < 0.01, as determined by Student’s *t*-test). (**H**) Fresh embryo weight of the WT and *rk1*. Values are means ± SD (*n* = 6; ** *p* < 0.01, as determined by Student’s *t*-test). (**I**) Two florets contain three anthers in the staminate spikelets of the WT. Scale bar = 2 mm. (**J**) Sessile spikelets typically contain three anthers, but pedicellate spikelets contain four anthers in the staminate spikelets of *rk1*. Scale bar = 2 mm. (**K**) Two spikelets with three anthers in a WT staminate spikelet, as observed in a paraffin cross section. Scale bar = 200 μm. (**L**) Pedicellate spikelet of *rk1* contains four anthers, as observed in a paraffin cross section. Scale bar = 200 μm. (**M**) Pollen viability staining conducted using TTC in WT anthers. Scale bar = 200 μm. (**N**) Pollen viability staining conducted using TTC in *rk1* anthers. Scale bar = 200 μm. (**O**) Pollen viability staining in the fourth anther conducted using TTC. Scale bar = 200 μm.

*rk1* is a spontaneous mutant isolated after multiple rounds of self-crossing from the progeny of the waxy corn cultivar ‘Xiaohuangnuo’. During vegetative growth, *rk1* plants are indistinguishable from their wild-type (WT) siblings. The averages and ranges of morphological and yield characteristics of the WT and *rk1* are given in Table S1. Closer examination of *rk1* ears revealed kernels with giant embryos facing the base of the ear, whereas WT kernels were oriented toward the tip of the ear (Figure 1A,B), representing the main difference between the WT and *rk1*. When we examined embryo orientations in cross sections of ears, embryos were clearly visible in the WT but not in *rk1* (Figure 1C,D).

We stained the embryos in longitudinal sections of WT and *rk1* grains with TTC (2,3,5-triphenyltetrazolium chloride) (Figure 1E,F). The embryo area and fresh embryo weight were larger in *rk1* vs. the WT (Figure 1G,H). We also counted the endosperm area and fresh endosperm weight, and the results show the endosperm area and fresh endosperm weight were both indeed smaller in *rk1* compared with WT (Figure S1). The grain crude protein and crude fat levels were significantly (*p*-value < 0.05) higher in *rk1* than in the WT (Table S1). In WT, a staminate spikelet contains one upper and one lower floret, and each floret contains three anthers (Figure 1I). In the staminate spikelets of *rk1*, partly pedicellate spikelets contain the fourth anthers (Figure 1J). We confirmed these results by observing paraffin sections (Figure 1K,L). We also performed TTC staining to visualize pollen particles in the anthers of WT (Figure 1M) and *rk1* spikelets (Figure 1N) and the fourth smaller anthers (Figure 1O), finding no significant difference in pollen viability.

### 2.2. rk1 Mapping

**Figure 2:**
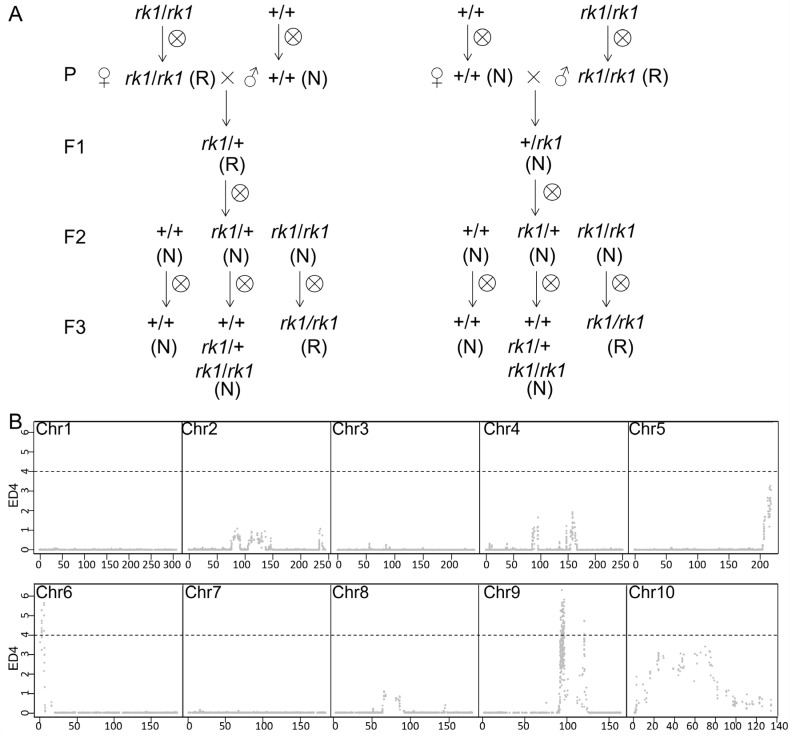
Genetic pattern and gene locations of *rk1*. (**A**) Conjectural genetic patterns of *rk1*. Genotypes of kernels are labelled as *rk1*/*rk1*, *rk1*/*+* and *+*/*+*, with the phenotypes labelled as N (normal kernels) and R (reversed kernels). (**B**) The scatter plots were exported from the BSA result. Distribution of ED^4^ across the 10 chromosomes. The abscissa number represents the physical coordinate (Mb).

We performed self-pollination of *rk1* and reciprocal crosses between *rk1* and the maize inbred line B73 to study the genetic inheritance and action of *rk1*. The reversed kernel phenotype of *rk1* can be inherited after self-crossing. We crossed *rk1* to B73 and crossed B73 to *rk1* to generate F_1_ hybrid (B73 × *rk1* and *rk1* × B73, respectively) kernels. When B73 was used as the female parent, B73 × *rk1* F_1_ plants produced normal kernels, whereas when *rk1* was used as the female parent, *rk1* × B73 F_1_ plants produced reversed kernels in the ear (Table S2). Self-pollination of both B73 × *rk1* and *rk1* × B73 F_1_ plants generated F_2_ kernels with normal phenotypes (Table S2), suggesting that *rk1* is a recessive mutant. Self-pollination of F_2_ plants generated segregating populations, with the number of ears producing normal kernels (F_3_) and the number of ears producing reversed kernels (F_3_) close to a 3:1 segregation ratio (165:67, χ^2^ = 2.074; 69:28, χ^2^ = 0.822 < χ^2^_0.05_ = 3.84; Table S2), suggesting that *rk1* harbors a single-gene mutation. According to the above results, we inferred the genetic mode of *rk1* as shown in Figure 2A. The phenotype of the offspring kernel is determined by the plant genotype. According to the phenotype of F3 kernels, the leaves of female plant F2 were selected as two mixing pools for DNA sequencing. BSA-Seq data analysis showed that the significant signal was mainly located at the 91.91–120.13 Mb (ED^4^ > 4.0) interval on chromosome 9, and a few signal points were located at 7.08–10.12 Mb on chromosome 6 (Figure 2B and Table S3). This indicates that chromosome 6 or 9 may be the location of the *rk1* gene.

### 2.3. UFM Differentiation Is Inhibited While LFM Differentiation Is Initiated in rk1

**Figure 3:**
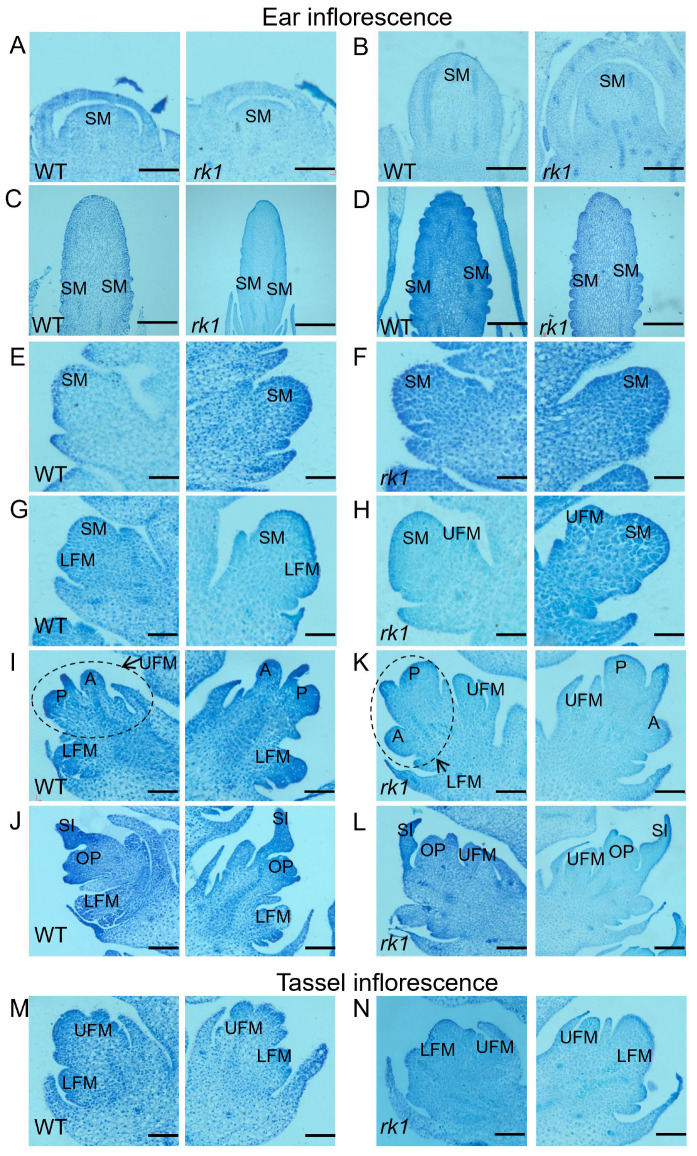
Paraffin section analysis of inflorescence differentiation in the WT and *rk1*. (**A**–**L**) Pistillate inflorescence development in the WT and *rk1*. (**M**,**N**) Staminate inflorescence development in the WT and *rk1*. Pistillate inflorescence development in the WT is shown in (**A**,**C**,**E**,**G**,**I,J**), and staminate inflorescence development is shown in (**M**). Pistillate inflorescence development in *rk1* is shown in (**B**,**D**,**F**,**H**,**K,L**), and staminate inflorescence development is shown in (**N**). The images on the left in (**E**–**L**) show the inflorescence to the left of the cone; the images on the right in (**E**–**L**) show the inflorescence to the right of the cone. Abbreviations: inflorescence meristem (IM), spikelet meristem (SM), lower floret meristem (LFM), upper floret meristem (UFM), pistil primordium (P), anther primordium (A), ovule primordium (OP), and silk (SI). Scale bars = 100 μm.

To define the developmental defects of *rk1*, we characterized the differentiation of inflorescences at different stages of development in both the WT and *rk1* via paraffin sectioning. There were no significant differences during the early stage of growth cone formation. In both lines, the inflorescence base formed a smooth IM (Figure 3A). During extension of the growth cone, the IM gradually elongated, again with no significant differences between lines (Figure 3B). During the spikelet differentiation stage, the SM formed gradually, with no significant differences between lines (Figure 3C,D).

During the inflorescence differentiation stage, the WT SM differentiated into an LFM and then developed into a UFM (Figure 3E). By contrast, each SM in *rk1* differentiated into a UFM, followed by an LFM (Figure 3F). During sexual organ development, the WT pistil primordium on the side of the cob formed a carpel (Figure 3G), while the floret meristem developed into the pistil primordium. Three WT anther primordia differentiated to encircle the pistil primordium, which then gradually grew upward to form silks and the ovule meristem (Figure 3H). By contrast, in the *rk1* pistil primordium, the epidermal protrusions on the side away from the cob formed a carpel (Figure 3I) before gradually forming silks and the ovule meristem (Figure 3J). Consistently, UFM differentiation was also inhibited in *rk1* during the staminate floret differentiation stage, whereas LFM differentiation was inhibited in the WT (Figure 3K,L). The development of *rk1* thus appeared abnormal during floret differentiation, with the lower florets developing first and the upper florets developing slowly until degradation.

### 2.4. The Order of UFM and LFM Development Is Reversed in rk1

**Figure 4:**
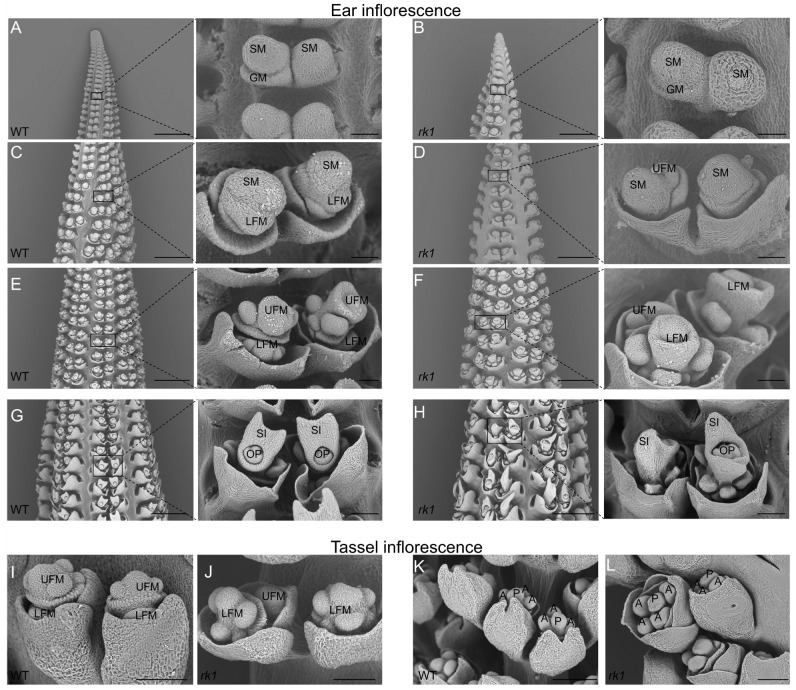
Inflorescence development in the WT and *rk1* revealed by scanning electron microscopy. (**A**–**H**) Pistillate inflorescence development in WT and *rk1*. The images on the right show higher magnification views of the boxed areas. Scale bars = 500 μm on the left and 50 μm in the magnified images. (**I**–**L**) Staminate inflorescence development in the WT and *rk1*. Pistillate inflorescence development in the WT is shown in (**A**,**C**,**E**,**G**), and staminate inflorescence development is shown in (**I**,**K**). Pistillate inflorescence development in *rk1* is shown in (**B**,**D**,**F**,**H**), and staminate inflorescence development is shown in (**J**,**L**). Scale bars = 100 μm. Abbreviations: spikelet meristem (SM), glume meristem (GM), lower floret meristem (LFM), upper floret meristem (UFM), pistil primordium (P), ovule primordium (OP), anther primordium (A), and silk (SI).

We performed scanning electron microscopy to clearly visualize the changes in the WT and *rk1* during different stages of inflorescence development. SPMs were arranged in rows initiated from the IM. The SPMs further developed to form two juxtaposed SMs, with no significant differences between the WT and *rk1* (Figure 4A,B). However, the LFM degenerated early and the UFM developed normally in the WT, whereas the sequence of UFM and LFM development was reversed in *rk1* (Figure 4C–F). In the WT, two carpels fused and elongated to form a silk (Figure 4G). However, several abnormal silks developed in *rk1*, which produced two nondirective silks, or a silk formed by a single carpel (Figure 4H). The staminate inflorescences were also reversed in *rk1* during the SM differentiation phase, as the UFM developed preferentially in the WT, but the LFM developed before the UFM in *rk1* (Figure 4I,J). We also detected some unusual changes to the staminate inflorescences of *rk1*, where some florets were accompanied by four anther primordia and accounted for 9.2%–13.9% (Figure 4K,L). 

**Figure 5:**
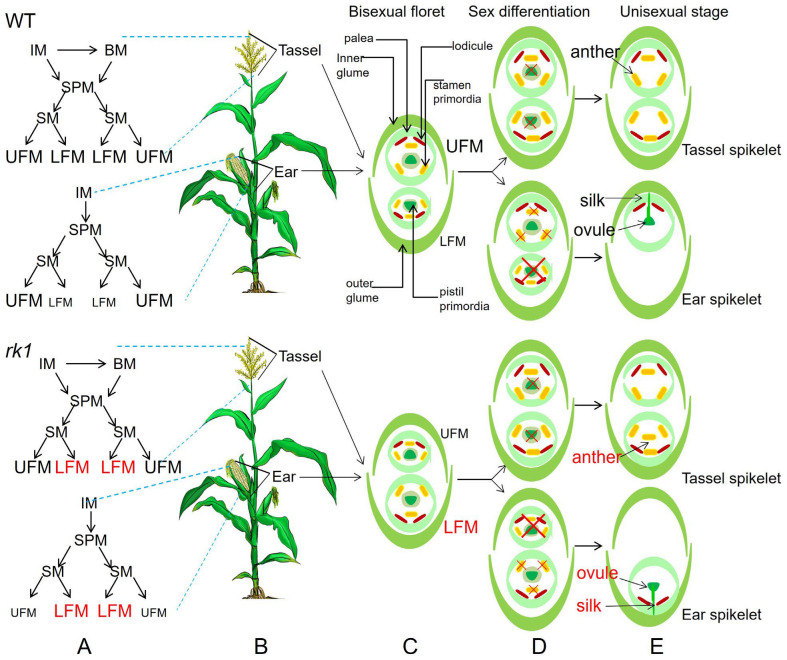
Proposed model for maize inflorescence structure and development in the WT and *rk1*. (**A**) Structural model of monoecious flowers in maize. (**B**) Diagram of monoecious flowers in maize. (**C**) Diagram of a spikelet prior to sex differentiation. (**D**) Diagram of a spikelet undergoing sex differentiation. (**E**) Diagram of a mature spikelet at the unisexual stage.

Based on our findings, we constructed a detailed model of WT and *rk1* flower development (Figure 5). This model is adapted from previous work [2,3,37]. Analysis of inflorescence initiation suggested that WT pistils abort in the tassels, but LFMs degenerate in the ears, whereas the order of UFM and LFM development is reversed in *rk1*, with the UFM degenerating in the ears of this mutant (Figure 5).

### 2.5. rk1 Influences the Expression of Genes Involved in Cell Differentiation, Organ Development, Phytohormone Responses, and Carbohydrate Metabolism

**Figure 6:**
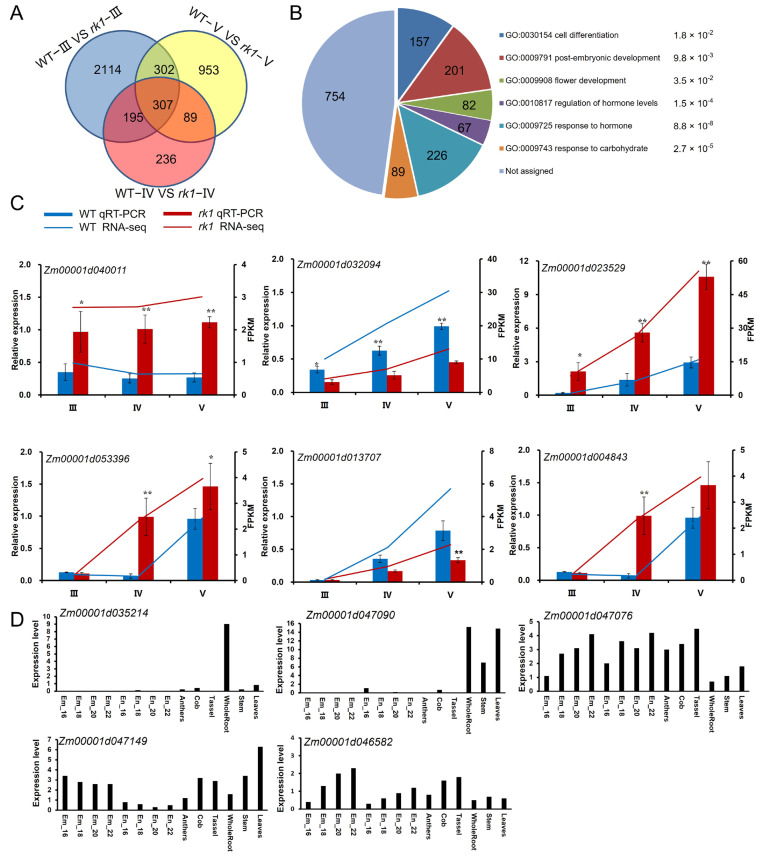
Transcriptome alterations in *rk1*. (**A**) Venn diagram showing the extent of overlap between DEGs from different groups. Spikelet differentiation period (III), floret differentiation period (IV), and sexual organ development period (V). (**B**) Most significantly enriched GO terms among DEGs between the WT and *rk1* based on RNA-seq analysis. The number of genes and the *p*-value for each GO term are shown. E indicates 10 raised to the indicated power in scientific notation. (**C**) RT-qPCR confirmation of the expression patterns of six representative DEGs in the WT and *rk1*. Values are means ± SD (*n* = 3; * *p* < 0.05; ** *p* < 0.01, as determined by Student’s *t*-test). (**D**) Expression of the deduced candidate genes for *rk1*. Em represents embryo, En represents endosperm and numbers represent days after pollination. Expression data were extracted from MaizeGDB qTeller.

Based on the reversed kernel development patterns described above (Figure 3 and Figure 4), we selected three stages of ear development for further analysis: the spikelet differentiation stage (III), floret differentiation stage (IV), and sexual organ formation stage (V). These three stages cover the emergence and appearance of the reversed kernel phenotype, as well as the critical determination period for the UFM and LFM. We identified differentially expressed genes (DEGs) between the WT and *rk1*, consisting of 2918 DEGs (1297 upregulated and 1621 downregulated) at stage III, 827 DEGs (320 upregulated and 507 downregulated) at stage IV, and 1652 DEGs (804 upregulated and 847 downregulated) at stage V (Figure 6A and Table S4). Significantly more genes were downregulated than upregulated during all three stages, based on comparisons between the WT and *rk1*. These results indicate that the transition of major biochemical processes along the developmental time axis of the inflorescence is at least partly attributed to highly coordinated transcript dynamics. In total, 307 DEGs were shared by the three stages of development in the WT vs. *rk1* (Figure 6A). These genes might play key roles in controlling inflorescence development.

Because this set of 307 genes was too small to conduct pathway enrichment analysis, we subjected the DEGs in *rk1* during the sexual organ formation stage to gene ontology (GO) functional annotation. Within these DEGs, 1576 could be functionally annotated by GO analysis (http://systemsbiology.cau.edu.cn/agriGOv2/; accessed on 27 March 2022). The six most significantly enriched GO terms were cell differentiation, post-embryonic development, flower development, regulation of phytohormone levels, response to phytohormone, and response to carbohydrate (Figure 6B and Table S5).

Notably, 440 DEGs were related to cell differentiation, post-embryonic development, or flower development, all of which play important roles in inflorescence differentiation and kernel development. The GO term response to carbohydrate stimulus (GO:0009743) was also enriched in the mutant DEGs. These results are consistent with the changes in nutrient reservoirs in *rk1* kernels (Table S1). In addition, 226 DEGs are associated with response to phytohormone (GO:0009725), including response to auxin (47 DEGs), ABA (85 DEGs), GA (31 DEGs), and JA (63 DEGs). Among these 226 DEGs, 115 are also involved in phytohormone-mediated signaling pathways (GO:0009755), including auxin, JA, GA, and ABA signaling pathways. In addition, 67 DEGs are associated with regulation of phytohormone levels (GO:0010817). Among these DEGs, 34 are involved in phytohormone biosynthesis (GO:0042446). These results indicate that phytohormone plays important roles in the inflorescence development of maize.

To verify the RNA-seq data, we performed RT-qPCR to measure the transcript levels of six selected DEGs. The results for these DEGs are similar to their fragments per kilobase of exon per million mapped fragments (FPKM) values (Figure 6C), confirming that the trends in the expression levels of the DEGs obtained by RNA-seq are reliable.

BSA-Seq data analysis showed that the significant signal was mainly located at the 91.91–120.13 Mb interval on chromosome 9, and some signal points were located at 7.08–10.12 Mb on chromosome 6 (Figure 2B and Table S3). RNA-seq analysis indicated that 307 DEGs were shared by the three stages of development in the WT vs. *rk1* (Figure 6A). Among the 307 DEGs, only *Zm00001d035214* was found in the chromosome 6 7.08–10.12 Mb interval and 4 genes (*Zm00001d046582*, *Zm00001d047076*, *Zm00001d047090*, *Zm00001d047149*) were found in the 91.91–120.13 Mb region on chromosome 9 (Table S4). Concerning the five genes, *Zm00001d035214* and *Zm00001d047090* were barely expressed in embryo, endosperm, anther, cob and tassel. Although *Zm00001d047149* and *Zm00001d046582* were expressed in all tissues, its expression level in embryo is extremely low, with only 0.3–0.8 (FPKM) of *Zm00001d047149* and 0.3–1.2 (FPKM) of *Zm00001d046582* (Figure 6D). Notably, the *Zm00001d047076* gene showed high expression in embryo, endosperm, anther, cob and tassel, the tissues of which were associated with inflorescence and kernel development (Figure 6D). *Zm00001d047076*, which encodes CAI-1 autoinducer sensor kinase/phosphatase cqsS isoform 1, was also expressed at a lower level in *rk1* than WT (Table S4). BSA-Seq and transcriptome analysis provide preliminary evidence that one of *Zm00001d047149*, *Zm00001d046582*, or *Zm00001d047076* is the probable candidate gene for *rk1*.

### 2.6. rk1 Affects Phytohormone Homeostasis

**Figure 7:**
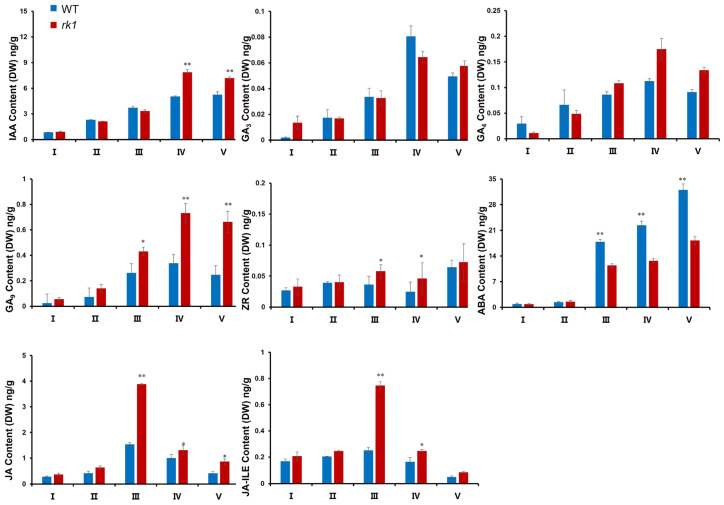
Phytohormone concentrations in WT and *rk1* inflorescences during different stages of development. The stages of inflorescence development are the growth cone unstretched period (I), growth cone elongation period (II), spikelet differentiation period (III), floret differentiation period (IV), and sexual organ development period (V). Values are means ± SD (*n* = 3; * *p* < 0.05; ** *p* < 0.01, as determined by Student’s *t*-test). IAA, indole-3-acetic acid; ZR, zeatin riboside; ABA, abscisic acid; GA, gibberellic acid; JA, jasmonic acid.

GO analysis suggested that the underlying mechanism responsible for reversed kernel development in *rk1* ears might directly involve phytohormonal regulation during pistillate inflorescence development, likely with synergistic effects. We thus quantified the contents of endogenous phytohormones in whole inflorescences throughout development by ultra-performance liquid chromatography–tandem mass spectrometry (UPLC-MS/MS). The endogenous IAA, GA_3_, GA_4_, GA_9_, ZR, JA, and JA-ILE contents of the WT and *rk1* tended to increase initially, followed by a decrease throughout the inflorescence differentiation stage (Figure 7). The IAA, GA_3_, GA_4_, and GA_9_ signals reached their peaks during the floret differentiation stage, but the peak for JA and JA-ILE occurred during the spikelet differentiation period. The ABA contents at different developmental stages were more variable compared with the IAA, GA_3_, GA_4_, GA_9_, and ZR contents, with the ABA contents tending to increase from the unstretched growth cone period to the sexual organ development period (Figure 7). IAA, GA9, ZR, JA, and JA-ILE levels increased significantly, while those of ABA decreased in *rk1* compared with the WT (*p* < 0.05 or *p* < 0.01; Figure 7).

**Figure 8:**
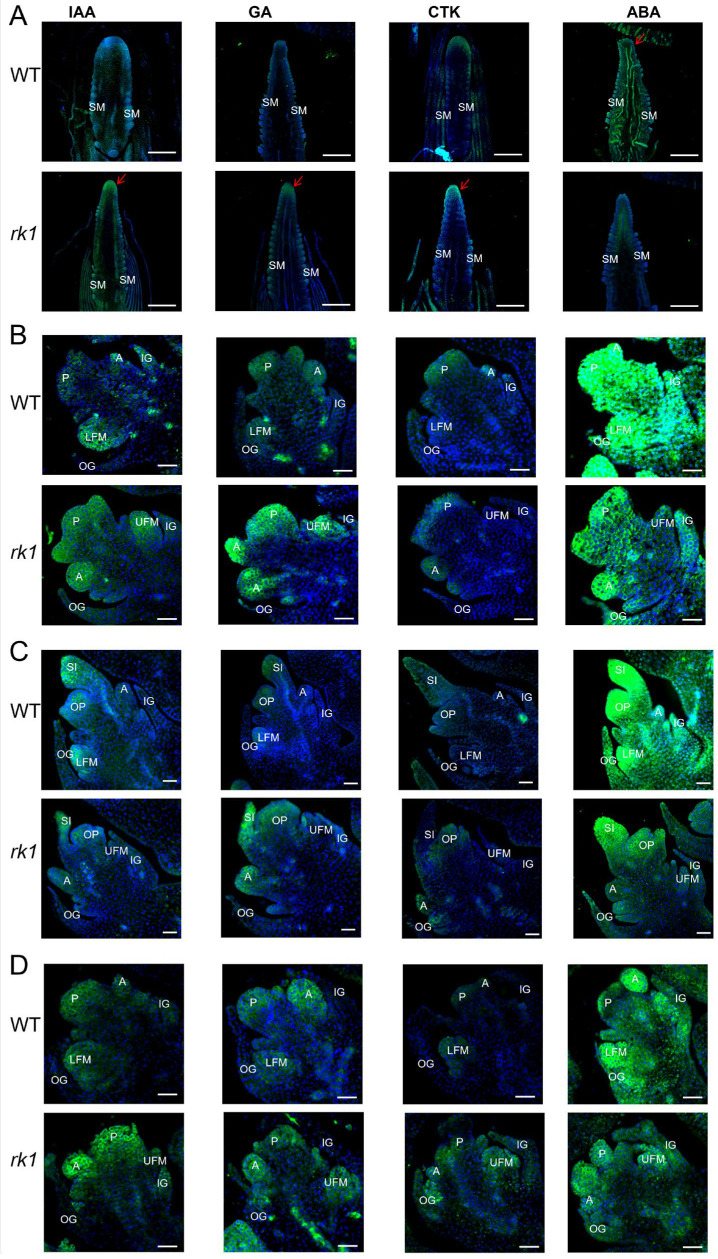
Immunofluorescence chemical localization of phytohormone distributions in inflorescences during different stages of development in the WT and *rk1*. (**A**) IAA, GA, CTK, and ABA immunohistochemistry localization signals in the WT and *rk1* during the pistillate spikelet differentiation stage. Scale bars = 200 μm. (**B**) IAA, GA, CTK, and ABA immunohistochemistry localization signals in the WT and *rk1* during the pistillate floret differentiation stage. Scale bars = 100 μm. (**C**) IAA, GA, CTK, and ABA immunohistochemistry localization signals in the WT and *rk1* during the pistillate organ formation stage. Scale bars = 100 μm. (**D**) IAA, GA, CTK, and ABA immunohistochemistry localization signals in the WT and *rk1* during staminate inflorescence development. Scale bars = 100 μm. Abbreviations: spikelet meristem (SM), inner glume (IG), outer glume (OG), lower floret meristem (LFM), upper floret meristem (UFM), pistil primordium (P), anther primordium (A), ovule primordium (OP), and silk (SI).

Dissection of young inflorescences followed by immunohistochemistry of individual phytohormones showed that the IAA, GA, CTK, and ABA localization signals differ significantly between the stages of pistillate inflorescence development. During spikelet initiation, the immunohistochemistry signals in the WT and *rk1* mainly distributed to the spikelet primordia. Moreover, the IAA, GA, and CTK signals in the spikelet apical meristem were stronger in *rk1* than in the WT, whereas the ABA signal intensity showed the opposite pattern (Figure 8A). 

At the floret differentiation stage, the IAA, GA, and CTK signals were mainly distributed in the UFM in the WT, whereas they were mainly distributed in the LFM in *rk1* (Figure 8B). However, the ABA signals were generally stronger in the WT than in *rk1* (Figure 8B). During sexual organ development, the IAA, GA, CTK, and ABA signals were generally weak in both the WT and *rk1*, but strong signals were distributed in the UFM in the WT and the LFM in *rk1*, and ABA signals were generally weaker in *rk1* than in the WT (Figure 8C). During the critical floret differentiation period in the staminate inflorescence, the IAA, GA, CTK, and ABA signals were similar to those observed in the pistillate inflorescence during the floret differentiation stage (Figure 8D).

## 3. Discussion

### 3.1. rk1 Is a Novel Mutant Impaired in Inflorescence Development

Normal ear and tassel development is critical for reproduction and grain yield in maize. *rk1* is the only maize mutant identified to date that specifically displays an altered order of UFM and LFM development. *rk1* exhibited a reversed kernel phenotype in the meristems and floral organs during inflorescence development, as well as producing giant embryos (Figure 1 and Table S1). The abnormal kernel direction in *rk1* was due to the preferential development of the LFM, whereas the UFM and anthers gradually degenerated during inflorescence development. The staminate inflorescences of *rk1* had similar morphological characters; they were oriented in the opposite directions in the LFM and UFM (Figure 3M–N and Figure 4I–L). 

Based on our results, we propose a model of the developmental sequence responsible for the formation of the reversed kernel phenotype of *rk1* (Figure 5). Previous studies have identified numerous mutants with abnormal grain direction, such as *ts4*, *ts6*, *ifa1*, *rgo1*, *wcr*, and *ids1* [8,9,10,17,38]. An unusual mutant, *thick tassel dwarf1* (*td1*), contains male florets with more stamens in the developing male inflorescence [39]. Other mutants with defective silk development, such as *silkless* (*sk1*), *zea agamous1* (*zag1*), *silky1* (*si1*), *branched silkless* (*bd1*), *required to maintain repression6* (*rmr6*), *bearded-ear* (*bde*), and *sterile tassel silky ear1* (*sts1*), possess extra silks or are silkless, often due to failed pistil abortion in the lower florets [37,40,41,42,43,44,45]. While *rk1* has reversed kernels with giant embryos, its phenotype does not completely resemble those of *ids1*, *rgo1*, *wcr*, *td1*, or *sk1* mutants, and its rows are more uniform in the ears. Notably, *rk1* and *rgo1*, *ids1* have different patterns of inheritance. The phenotype of *rgo1* and *ids1* is determined jointly by the genotype of the parents, whereas the phenotype of *rk1* is determined by the plant genotype. In fact, the genotype of the seed does not determine the orientation of the seed, but rather the genotype of the plant determine the development of upper and lower floral meristem for the kernel orientation in *rk1*. The upper and lower floral meristem development in *rk1* is consistent with Mendel’s genetic law. In addition, *rgo1* mutants are similar to wild types except that each SM initiates two UFMs before converting into an FM [10]. In *rgo1* spikelets, the lowest flower aborts and the two upper flowers develop. The two upper flowers produce a reversed kernel and a normal orientation kernel, respectively [10]. *ids1* mutants delay the SM to FM conversion, resulting in the production of extra florets [9,10]. By contrast, each SM in *rk1* differentiated into a UFM, followed by an LFM. The abnormal kernel direction in *rk1* was due to the preferential development of the LFM, whereas the UFM gradually degenerated during inflorescence development. The lower flower produced a reversed kernel. In addition, we looked up mutants associated with inflorescence development [46], and found no reports about the function of the order of UFM and LFM development in maize. In this study, we demonstrated that the reverse order of UFM and LFM development can lead to the production of reversed kernels with giant embryos, which has not previously been reported. The *rk1* mutant could be employed to investigate how maize inflorescences develop and to increase the available germplasm for the nutritional improvement of maize due to its giant embryos.

### 3.2. rk1 Influences Organ Differentiation, Phytohormone Homeostasis, and Nutrient Contents

Phytohormonal homeostasis is tightly integrated in order to establish robust systems that allow plant growth to adapt to continuous inputs from the environment. Improper endogenous phytohormone levels can disrupt primordium initiation during inflorescence development. We conducted immunohistochemical localization to determine the in-situ distributions and levels of phytohormones during inflorescence development in the WT and *rk1* (Figure 8). This approach has been used to analyze other plant hormones, such as IAA in maize and wheat (*Triticum aestivum*) [47], GA in buffalo grass (*Bouteloua dactyloides*) [48], CTK in tobacco (*Nicotiana tabacum*) [49], and ABA in wheat [50]. The IAA, GA, CTK, and ABA levels differed significantly between the WT and *rk1* during different developmental stages, such as when the styles of the pistillate florets began stigmatic tissue differentiation. However, after the development of SMs, the intensity and distribution of the IAA, GA, and CTK immunofluorescence signals were brighter in the UFM than the LFM in the WT relative to *rk1* (Figure 8). Although the results of IAA detection in WT and *rk1* correspond with the expected zone of auxin maxima, the IAA detection of *rk1* was significantly different from those of WT in the stages of pistillate inflorescence development and tissue sites. Those differences may be an important factor affecting the development of upper and lower floral meristem.

IAA and GA were previously associated with inflorescence development in maize, as the position of LFM initiation is regulated by polar IAA transport [51]. However, GA plays the opposite role, as its local biosynthesis is thought to suppress stamen development [52], and GA promotes pistil development in tassels [53]. Thus, we hypothesize that IAA, GA, and CTK play major roles in UFM development in the WT pistillate inflorescence, as well as the *rk1* LFM, and that changes in phytohormonal homeostasis may be associated with abnormal differentiation of the gynoecium. 

We detected bright fluorescent signals for ABA from the spikelet differentiation stage until the sexual organ formation stage in the pistillate inflorescences of the WT and *rk1*, as well as the floret differentiation stage in staminate inflorescences (Figure 8), thereby demonstrating that high ABA levels are maintained during the sexual organ formation period. Previous studies have demonstrated that the sexual organ fate of the maize inflorescence is regulated by JA and GA, where the JA-mediated signaling pathway is responsible for pistil abortion and stamen development and vice versa [52,54]. In addition, Luo has determined that the homeostasis of JA and GA affects the sex reversal phenotype of the *si3* mutant [55]. 

In the present study, we examined the responses of various genes to JA via RNA-seq (Table S5). The reciprocal regulation among IAA, GA, CTK, and ABA pathways is important for the regulatory network of pistil or stamen development and for determining pistil or stamen fate [2]. Perhaps the relative homeostasis of IAA, CTK, GA, ABA, or JA helps promote inflorescence development during pistil or stamen development.

GO analysis shows that 440 DEGs function in cell differentiation, post-embryonic development, or flower development (Figure 6B). During the inflorescence differentiation stage, the WT SM differentiated into an LFM and then developed into a UFM (Figure 3), whereas in *rk1*, each SM differentiated into a UFM and then formed an LFM (Figure 3). These DEGs may play important roles in the inflorescence differentiation stage. 

We stained longitudinal sections of kernels with TTC, showing that the embryos are much larger in *rk1* vs. the WT (Figure 1E–H). The fresh embryo weight, crude protein, and crude fat contents were higher in *rk1* than in the WT, indicating that the nutrient contents are higher in *rk1* kernels (Table S1). Moreover, GO analysis indicated that the GO term response to carbohydrate stimulus (GO:0009743) is also enriched among the DEGs. Thus, *rk1* may represent a valuable resource for improving the nutritional quality of waxy corn.

In summary, we developed a maize inbred line harboring the mutation *rk1*, with inverted giant embryos, which we confirmed by morphological, histocytological, and RNA-seq analyses. Based on our findings, we developed a model of the highly complex regulatory mechanism that determines the phenotype of *rk1* (Figure S2). *rk1* represents a useful tool for studying flower development in maize. Identifying the causal gene in *rk1* could provide new insights into the regulatory networks involved in inflorescence development.

## 4. Materials and Methods

### 4.1. Plant Materials

*rk1* is a spontaneous maize mutant isolated after multiple rounds of self-crossing from the progeny of a hybrid plant derived from the local Chinese waxy corn cultivar ‘Xiaohuangnuo’. Normal and mutant seeds were propagated for multiple generations. Normal seeds whose offspring no longer separated were used as the wild type (WT) in subsequent experiments. *rk1* was crossed with maize inbred line B73, and B73 was crossed with *rk1* to produce reciprocal segregating populations for genetic analysis. These plant materials are preserved at the Specialty Corn Institute, College of Agronomy, Shenyang Agricultural University, Shenyang, Liaoning, China.

### 4.2. Morphological Evaluation

During the sowing seasons of 2017, 2018, and 2019, a randomized complete block design with two replicates was used to evaluate the major agronomic traits of the WT and *rk1* (i.e., plant height, ear height, ear length, row number per ear, kernels per row, 100-grain weight, fresh kernel weight, fresh embryo weight, and pollen viability). After harvesting, 50 plants were examined from each plot to evaluate the traits. Grain characters (especially fresh embryo weights) were measured 20 days after pollination in the WT and *rk1* as described by Zhang [56]. All parameters were averaged, and each trait was compared using a two-tailed Student’s *t*-test. TTC (2,3,5-triphenyltetrazolium chloride) staining was performed according to Zhang [56]. Longitudinal sections of the embryos and the vitality of pollen particles in the WT and *rk1* were examined after TTC staining under a stereomicroscope (ZEISS Axio Zoom.V16, Oberkochen, Germany), captured with a Photometrics SenSys CCD camera, and processed using ZEN 2012 (Blue Edition, 1.1.2.0) imaging software.

### 4.3. Paraffin Sectioning

Paraffin sectioning was performed as described by Kaplinsky and Freeling [10], with some modifications. The sections in paraffin wax were cut with a manual microtome (Leica RM2235, Wetzlar, Germany) and stained with 0.025% (*w*/*v*) toluidine blue. Dynamic inflorescence differentiation was observed in the WT and *rk1* at different developmental stages under a stereomicroscope (ZEISS Axio Zoom.V16, Oberkochen, Germany), captured with a Photometrics SenSys CCD camera, and processed using ZEN 2 (Blue Edition) imaging software.

### 4.4. Scanning Electron Microscopy

The ears and tassels of the WT and *rk1* at different developmental stages were fixed in formalin–acetic acid–alcohol solution (5 mL 38% (*w*/*v*) formaldehyde, 5 mL glacial acetic acid, and 90 mL 70% (*v*/*v*) ethanol) for 24 h. The samples were dehydrated with different concentrations of ethanol and tert-butanol and freeze-dried (Vacuum Device, VFD-30, Canton, TX, USA). The samples were fixed on a coating machine (Hitachi, MSP-2S, Tokyo, Japan), dried, sprayed with gold particles, and examined to visualize changes in the different inflorescence features of the WT and *rk1* during various developmental stages under a scanning electron microscope (Hitachi, TM-3030, Tokyo, Japan). 

### 4.5. RNA Isolation, Library Construction, and RNA Sequencing

Total RNA was extracted from all samples using TRIzol reagent (Invitrogen, Carlsbad, CA, USA). Degradation and contamination of RNA with genomic DNA were determined by running an aliquot on 1% (*w*/*v*) agarose gels. RNA quality was evaluated with a Bioanalyzer 2100 system (Agilent Technologies, Santa Clara, CA, USA). mRNA libraries were constructed and sequenced using an Illumina HiSeq 2500 platform (Illumina, San Diego, CA, USA). Paired-end 150 libraries were sequenced using next-generation sequencing by Personal Biotechnology Co. Ltd. (Shanghai, China).

### 4.6. Bulk Segregant Analysis Sequencing (BSA-Seq)

DNA was extracted from the fresh leaves of plants of the F2 population. Two pools were constructed by mixing equal amounts of DNA from 30 F2 plants carrying normal kernels and 30 F2 plants carrying reversed kernels, respectively. Genome resequencing was performed with Illumina HiSeq Xten under paired-end 150 bp mode and 20× coverage for each pool. After removing low-quality reads and adapter sequences, the clean reads were aligned to the maize B73 reference genome (AGP v.4) using BWA software (0.7.17) [57,58]. Picard software (1.119) was then employed to fix mates, sort read groups, and remove PCR duplicates from the bam files. SNPs were called with HaplotypeCaller module from GATK [57,59] and converted to VCF files for both low and high pools. The SNPs with a low coverage < 3, and minimum variant frequency < 0.05 were excluded. First, the ED for each SNP, described as ED_SNP_, was calculated:

$${\mathrm{ED}}_{\mathrm{SNP}}=\sqrt{{\left(Amut-Awt\right)}^{2}+{\left(Cmut-Cwt\right)}^{2}+{\left(Gmut-Gwt\right)}^{2}+{\left(Tmut-Twt\right)}^{2}}$$

where each letter (*A*, *C*, *G*, *T*) corresponds to the frequency of its corresponding DNA nucleotide. ED is the sum of 100 ED_SNP_ values within a window of 100 consecutive SNPs. ED4 was then calculated by raising ED to the fourth power [60].

### 4.7. Quantitative RT-PCR

Quantitative RT-PCR (RT-qPCR) was performed to verify the reliability of the sequencing data by selecting differentially expressed genes (DEGs). qPCR was performed using the ChamQ^TM^ Universal SYBR^®^ qPCR Master Mix on a Stratagene Mx3000P system (Agilent Technologies). The gene amplification program and relative quantification method have been described previously [55]. The primers are described in Table S6.

### 4.8. Immunolabeling 

For immunolabeling, tissue sections acquired by paraffin sectioning were placed in citric acid antigen repair buffer (pH 6.0, Servicebio) for antigen retrieval in a microwave oven. The sections were treated with primary rabbit polyclonal antibodies (anti-IAA, anti-GA, anti-CTK, and anti-ABA) (shay-bio) diluted 100-fold in phosphate buffered saline (PBS) (pH 7.4) and incubated in a humid chamber at 4 °C overnight. The slides were washed three times in PBS (pH 7.4) for 5 min each time, treated with Alexa Fluor 488-labeled goat anti-rabbit (as the secondary antibody; Servicebio, Wuhan, China) diluted 400-fold in PBS (pH 7.4), and incubated for 50 min at room temperature. After washing the slices three times with PBS, 6-diamidino-2-phenylindole (DAPI, Servicebio) stain was added dropwise, and the samples were incubated for 10 min at room temperature. Control reactions were performed without the primary antibodies. Images were obtained under an upright fluorescence microscope (NIKON ECLIPSE C1, Minato ku, Japan) and processed using NIKON DS-U3 (Ver.1.00) imaging software [61].

### 4.9. Extraction and Evaluation of Endogenous Phytohormones by Liquid Chromatography–Mass Spectrometry (LC-MS)

The pistil and stamen inflorescences of WT and *rk1* plants at different developmental stages were processed using a modified version of the method described by Zhang [62]. All standards for plant hormones were supplied by Sigma. SPSS 6.0 software was used to conduct one-way analysis of variance and Duncan’s new multiple range test to determine significant differences (*p* < 0.05, *p* < 0.01) between the results obtained for the WT and *rk1* and to conduct *t*-tests of independent samples to examine the effects of variety and determine correlations.

## 5. Conclusions

We characterized the *rk1* mutant in which the reverse order of UFM and LFM development results in reversed kernels. The genetic inheritance showed that *rk1* is a monogenic mutation. The phenotype of offspring kernel direction is determined by the plant genotype. BSA-Seq and transcriptome analysis provide preliminary evidence that one of *Zm00001d047149*, *Zm00001d046582*, or *Zm00001d047076* is the probable candidate gene for *rk1*. In order to further identify candidate genes of *RK1*, the candidate regions can be confirmed by mapping cloning using polymorphic markers, and candidate genes can be analyzed by gene sequencing and in situ hybridization. Transcriptome analysis revealed differentially expressed genes in *rk1* related to cell differentiation, organ development, phytohormonal responses, and carbohydrate metabolism. Changes in phytohormone homeostasis during inflorescence differentiation and pistillate inflorescence development are likely associated with the mutant *rk1* phenotype.

## Data Availability

All data analyzed during this study are provided in this published article and Supplementary Materials. The RNA-seq data are available from the National Center for Biotechnology Information Gene Expression Omnibus (www.ncbi.nlm.nih.gov/geo accessed on 4 April 2022) under the series entry PRJNA827497. All the raw sequencing data for BSA can be found in the National Genomics Data Center (https://bigd.big.ac.cn/ accessed on 1 April 2023) under Bioproject no. CRA010493.

## Supplementary Materials

The following supporting information can be downloaded at: https://www.mdpi.com/article/10.3390/ijms241310728/s1.
