# Supplementary material for: BSA-Seq and Transcriptomic Analysis Provide Candidate Genes Associated with Inflorescence Architecture and Kernel Orientation by Phytohormone Homeostasis in Maize
Source: Int J Mol Sci. 2023 Jun 27;24(13):10728. doi: 10.3390/ijms241310728 (PMC10341581; doi:10.3390/ijms241310728)
Supplement: Supplementary file 1 [file ijms-24-10728-s001.zip › ijms-2433114-supplementary/Supplementary materials/Supplementary Figure.pdf]

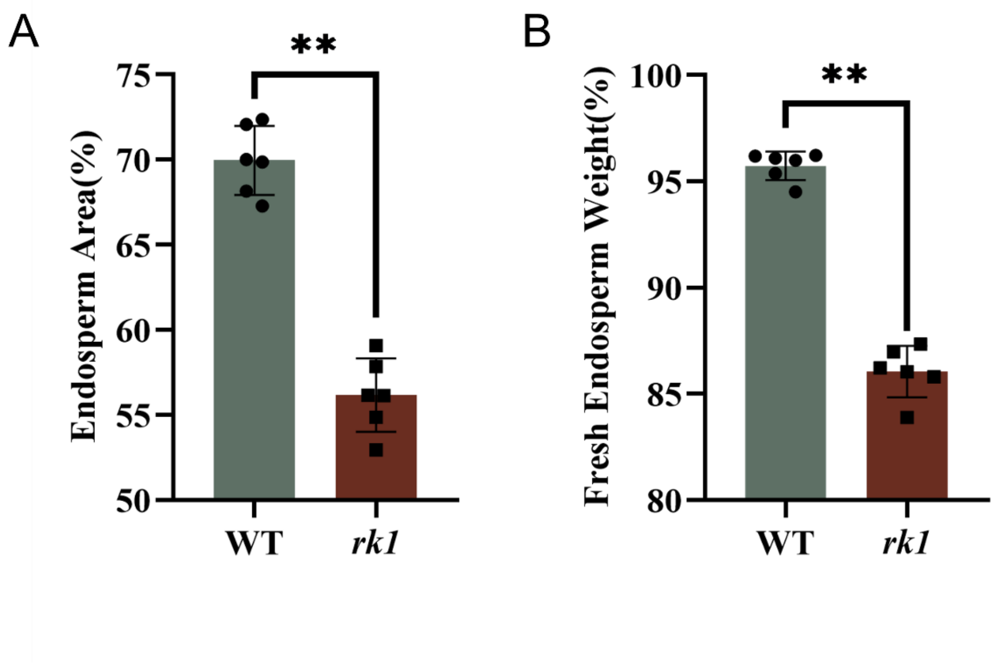

Figure S1. The endosperm area and fresh embryo weight in the WT and *rk1*. (A) Area of the endosperm in WT and *rk1* kernels. Values are means ± SD (n = 6; $^{**}P < 0.01$, as determined by Student's *t*-test). (B) Fresh endosperm weight of the WT and *rk1*. Values are means ± SD (n = 6; $^{**}P < 0.01$, as determined by Student's *t*-test).

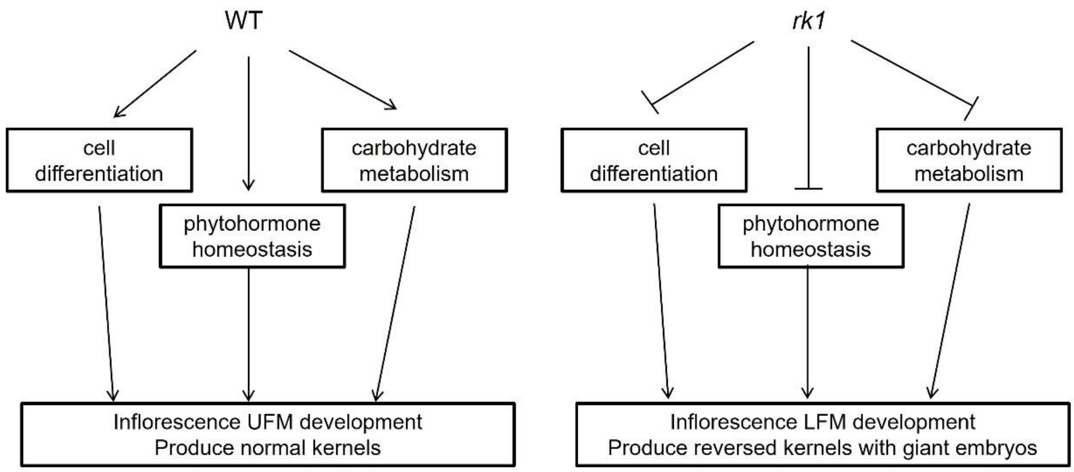

Figure S2. The deduced model of the phenotypic consequences of the *rk1* mutation. The deduced model shows the highly complex regulatory mechanism that determines the phenotype of *rk1* vs. the WT.
